# Complete genome sequences of 17 *Salmonella enterica* serovar Schwarzengrund isolates carrying an IncFIB-IncFIC (FII) fusion plasmid

**DOI:** 10.1128/mra.01062-23

**Published:** 2024-01-17

**Authors:** Danielle Sopovski, Seth Commichaux, Shaohua Zhao, Christopher J. Grim, Steven L. Foley, Bijay K. Khajanchi

**Affiliations:** 1 National Center for Toxicological Research, U.S. Food and Drug Administration, Jefferson, Arkansas, USA; 2 Center for Food Safety and Applied Nutrition, Office of Applied Research and Safety Assessment, U.S. Food and Drug Administration, Laurel, Maryland, USA; 3 Center for Veterinary Medicine, Office of Applied Science, U.S. Food and Drug Administration, Laurel, Maryland, USA; 4 Center for Food Safety and Applied Nutrition, Office of Regulatory Science, U.S. Food and Drug Administration, College Park, Maryland, USA; Wellesley College Department of Biological Sciences, USA

**Keywords:** *Salmonella S*chwarzengrund, fusion plasmid, IncFIB-IncFIC (FII), nanopore

## Abstract

Seventeen *Salmonella enterica* serovar Schwarzengrund isolates from chicken (*n* = 9) and clinical samples including stool (*n* = 6), urine (*n* = 1), and gallbladder (*n* = 1) were sequenced and found to carry an IncFIB-IncFIC (FII) fusion plasmid of approximately 145 Kb. This information provides reference genomic data for comparative studies of *S*. Schwarzengrund pathogenicity and plasmid genetics.

## ANNOUNCEMENT

There has been an increasing occurrence of global foodborne illnesses caused by *Salmonella enterica* serovar Schwarzengrund (1). *S*. Schwarzengrund can infect both humans and animals and has caused several outbreaks (2).

A broad range of plasmid incompatibility groups (Inc) have been observed in *Salmonella* serovars (3–6). These plasmids can contribute to the horizontal transfer of antimicrobial resistance and virulence genes (6, 7).

*S*. Schwarzengrund isolates were cultured and identified using standard microbiological methods (8). Briefly, samples were plated on Hektoen enteric agar, *Salmonella Shigella* agar, and enrichment broths [Becton Dickinson and Company (BD), Franklin Lakes, NJ, USA] and then incubated at 35°C for 18–24 hours. Presumptive lactose-negative, H_2_S-positive colonies were subsequently inoculated in motility-indole-lysine agar, triple sugar iron agar, and urea agar plate (BD). Identification was confirmed using matrix assisted laser desorption ionization-time of flight mass spectrometry (MALDI-TOF MS).

A total of 17 isolates were sequenced using long-read Oxford Nanopore Technology (ONT). All 17 isolates were also sequenced using Illumina technology, which included two that were sequenced in this report and 15 that were sequenced previously (Table 1). Isolates were sequenced independently using separate DNA extractions. For Illumina sequencing, total bacterial DNA was extracted using a DNeasy Blood and Tissue kit (Qiagen, Valencia, CA, USA), and DNA sequencing libraries were constructed using 1 ng of DNA from each sample using the Nextera XT DNA library preparation kit (Illumina, San Diego, CA, USA). Samples were multiplexed using a unique combination of two indexes of Nextera XT Index Kit. DNA samples were diluted, denatured, and loaded on an Illumina MiSeq and 2 × 250 bp paired end sequencing was performed. Trimming and *de novo* assembly were performed using CLC Genomics Workbench (version 9, Qiagen, Germantown, MD, USA).

**TABLE 1:** Complete genome analysis of 17 *Salmonella* Schwarzengrund isolates from food and clinical sources

Sample ID	Biosample	States	Source	Year	Number of contigs	Chromosome length (bp)	ONT number of reads	ONT reads N50 (bp)	Depth of chromosome coverage	Accession number for hybrid assembly	SRA accession for Illumina sequencing	SRA accession for Oxford Nanopore sequencing
CVM-5	SAMN35361192	GA	Chicken Wings	2013	4	4,866,971	22,866	25,649	31.00	CP130126-CP130129	SRR2566870 ^*a*^	SRR26617622
CVM-7	SAMN35361193	GA	Chicken Breast	2013	8	4,770,521	28,685	24,160	35.00	CP130118-CP130125	SRR2566919 ^*a*^	SRR26617621
CVM-10	SAMN35361194	GA	Chicken Breast	2013	3	4,852,253	20,963	23,526	23.14	CP130115-CP130117	SRR2567043 ^*a*^	SRR26617613
CVM-11	SAMN35361195	GA	Chicken Breast	2013	3	4,852,220	23,115	23,705	29.74	CP130112-CP130112	SRR2567044 ^*a*^	SRR26617612
CVM-13	SAMN35361196	NM	Chicken Breast	2013	3	4,818,423	10,832	31,520	27.42	CP130109-CP130111	SRR2567116 ^*a*^	SRR26617611
CVM-14	SAMN35361197	GA	Chicken Wings	2013	3	4,859,311	21,739	23,102	25.58	CP130106-CP130108	SRR2567150 ^*a*^	SRR26617610
CVM-15	SAMN35361198	GA	Chicken Breast	2013	3	4,853,146	16,658	24,879	20.39	CP130103-CP130105	SRR2567152 ^*a*^	SRR26617609
CVM-16	SAMN35361199	GA	Chicken Breast	2013	4	4,823,778	253,735	19,103	397.31	CP130099-CP130102	SRR2567160 ^*a*^	SRR26617608
CVM-17	SAMN35361200	NM	Chicken Breast	2013	3	4,864,549	224,895	24,335	393.86	CP130096-CP130098	SRR2567182 ^*a*^	SRR26617607
MDH-29	SAMN35361201	MN	Urine	2017	5	4,861,538	12,814	33,739	31.89	CP130091-CP130095	SRR6050821 ^*a*^	SRR26617606
WLSH-3	SAMN35361202	WI	Stool	2017	5	4,851,881	29,473	25,237	29.14	CP130086-CP130090	SRR5908531 ^*a*^	SRR26617620
WLSH-7	SAMN35361203	WI	Gallbladder	2017	3	4,854,947	19,729	24,471	21.48	CP130083-CP130085	SRR6345645 ^*a*^	SRR26617619
WLSH-13	SAMN35361204	WI	Stool	2016	4	4,871,194	15,681	33,854	33.35	CP130274-CP130277	SRR10206152 ^*a*^	SRR26617618
WLSH-25	SAMN35361205	WI	Stool	2013	7	4,850,894	359,918	17,367	346.32	CP130076-CP130082	SRR10206139	SRR26617617
WLSH-26	SAMN35361206	WI	Stool	2013	3	4,850,917	368,653	24,270	392.24	CP130073-CP130075	SRR10206151	SRR26617616
MD-3	SAMN35361207	MD	Stool	2016	3	4,863,192	446,772	19,037	430.46	CP130070-CP130072	SRR5201368 ^*a*^	SRR26617615
MD-4	SAMN35361208	MD	Stool	2016	4	4,861,906	432,018	19,196	418.59	CP130270-CP130273	SRR5238054 ^*a*^	SRR26617614

^*a*^ Sequencing raw data submitted by other investigators.

For ONT sequencing, the 1D native barcoding genomic DNA long read selection protocol was used with the SQK-LSK109 kit (Oxford Nanopore, Oxford, UK) as described earlier (9). Briefly, 1 µg of DNA was subjected to end repair and dA-tailing using NEBNext Ultra II End Repair/dA-Tailing module (New England Biolabs). End-prepped DNA fragments were barcoded using the EXP-NBD104 and EXP-NBD114 kits (ONT, UK). Equimolar amounts of each barcoded sample were pooled together, adapters ligated, and the resulting library pools sequenced on a MinION device using a FLO-MIN106 (R9.4.1) flow cell for 48 h.

The nanopore reads were trimmed and filtered using NanoFilt (v2.3.0) (10). The combined short and long read sequences were assembled by hybrid assembly using UniCycler (v0.4.8) (11). Platon (v1.6) (12) was used to annotate the assembly contigs for plasmids. Final annotation was performed using the NCBI Prokaryotic Genome Annotation Pipeline (PGAP v5.0) (13). All analyses were performed using default parameters for the software.

The mean of genome size was approximately 4.8 Mbp. A ~145 Kb fusion IncFIB-IncFIC (FII) plasmid was identified in all the isolates. Some isolates carried additional plasmids. The depth of coverage ranged from 20× to 430× (Table 1).

## Data Availability

This complete genome project has been deposited at GenBank under BioProject PRJNA954313 and the accession numbers for hybrid assembly and the SRA accessions for raw data are listed in Table 1.
